# A Chinese patent medicine’s long-term efficacy on non-dialysis patients with CKD stages 3–5: a retrospective cohort study

**DOI:** 10.3389/fphar.2024.1379338

**Published:** 2024-04-26

**Authors:** Hui-Fen Chen, Yu-Jie Lin, Yan Han, Xian-Long Zhang, Ruo-Bing Wang, Jun-Hui Chen, Ying Pi, Li-Zhe Fu, Fang Tang, Xi-Na Jie, Xiao-Na Tang, Xu-Sheng Liu, Yi-Fan Wu

**Affiliations:** ^1^ The Second Clinical College of Guangzhou University of Chinese Medicine, Guangzhou, China; ^2^ Renal Division, The Second Affiliated Hospital of Guangzhou University of Chinese Medicine (Guangdong Provincial Hospital of Chinese Medicine), Guangzhou, China; ^3^ Bao’an Traditional Chinese Medicine Hospital, Guangzhou University of Chinese Medicine, Shenzhen, China; ^4^ Chronic Disease Management Outpatient Clinic, The Second Affiliated Hospital of Guangzhou University of Chinese Medicine (Guangdong Provincial Hospital of Chinese Medicine), Guangzhou, China

**Keywords:** chronic kidney disease, long-term efficacy, Chinese patent medicine, longitudinal analyze, Niaoduqing granules

## Abstract

**Background:**

Chinese patent medicine is commonly used in China as an important treatment mechanism to thwart the progression of chronic kidney disease (CKD) stages 3–5, among which Niaoduqing granules are a representative Chinese patent medicine; however, its long-term efficacy on CKD prognosis remains unclear.

**Methods:**

Patients were grouped according to Niaoduqing granule prescription duration (non-Niaoduqing granule (non-NDQ) group vs Niaoduqing granule (NDQ) group). Serum creatinine (SCr) variation was compared using a generalized linear mixed model (GLMM). Multivariate Cox regression models were constructed, adjusting for confounding factors, to explore the risk of composite outcomes (receiving renal replacement therapy (RRT) or having an estimated glomerular filtration rate (eGFR)<5 mL/min/1.73 m^2^, ≥50% decline in the eGFR from the baseline, and doubling of SCr) in individuals consuming Niaoduqing granules.

**Results:**

A total of 1,271 patients were included, with a median follow-up duration of 29.71 (12.10, 56.07) months. The mean SCr Z-scores for the non-NDQ group and NDQ group were −0.175 and 0.153, respectively, at baseline (*p* = 0.015). The coefficients of the NDQ group from visit 1 to visit 5 were −0.207 (95% CI: −0.346, −0.068, *p* = 0.004), −0.214 (95% CI: 0.389, −0.039, *p* = 0.017), −0.324 (95% CI: 0.538, −0.109, *p* = 0.003), −0.502 (95% CI: 0.761, −0.243, *p* = 0.000), and −0.252 (95% CI: 0.569, 0.065, *p* = 0.119), respectively. The survival probability was significantly higher in the NDQ group (*p* = 0.0039). Taking Niaoduqing granules was a significant protective factor for thwarting disease progression (model 1: HR 0.654 (95% CI 0.489–0.875, *p* = 0.004); model 2: HR 0.646 (95% CI 0.476, 0.877, *p* = 0.005); and model 3: HR 0.602 (95% CI 0.442, 0.820, *p* = 0.001)).

**Conclusion:**

The long-term use of Niaoduqing granules improved SCr variation and lowered the risk of CKD progression by 39.8%.

## 1 Introduction

Chronic kidney disease (CKD) is defined as having a glomerular filtration rate (GFR) less than 60 mL/min/1.73 m^2^ with abnormalities in kidney structure biomarkers and/or function persisting for at least 3 months (National Kidney Foundation, 2002). It is a global health problem affecting 7.0%–34.3% of adults (Liyanage et al., 2022). Patients with stages 3–5 CKD experience more acute kidney impairment and worse outcomes. Renal replacement therapy (RRT) is required once a patient has progressed to end-stage kidney disease (ESRD), exerting a heavy burden on individuals, their families, and even society at large.

Therapies for CKD stages 3–5 consist of lifestyle adjustment, etiology treatment, and complication prevention. Medications such as angiotensin-converting enzyme inhibitors/angiotensin receptor blockers (ACEIs/ARBs) and sodium-glucose cotransporter-2 inhibitors (SGLT2i) (KDIGO, 2021b) are routinely used to thwart disease progression. Traditional Chinese medicine (TCM) holds that CKD often arises from chronic nephropathy, diabetic nephropathy, and other syndromes. The primary etiologies underlying CKD are multifaceted and can be attributed to congenital deficiencies, unhealthy dietary habits, excessive exertion, external pathogenic influences, and emotional imbalances. Prolonged illness will be attributed to the deficiency of spleen–kidney *qi,* deficiency of both *qi* and *yin,* and accumulation of pathological factors such as dampness and blood stagnation, leading to a more complex and challenging illness to treat. In China, various Chinese patent medicines have become important treatments to thwart the progression of CKD stages 3–5 (Standardization project group, 2021; Wang et al., 2023). Niaoduqing granules are a representative Chinese patent medicine and have been used for years. They contain the following ingredients: *Radix Astragali (黄芪 huánɡ qí),* *Codonopsis pilosula* (*党参 dǎnɡ shēn*)*, Radix Polygoni Multiflori Preparata (制何首乌 zhì hé shǒu wū)*, *Radix et Rhizoma Rhei (生大黄 shēnɡ dà huánɡ)*, *Rhizoma Atractylodis macrocephalae (白术 bái zhú)*, *Wolfiporia extensa* (*茯苓 fú línɡ*), *Plantago asiatica* ( *车前草 chē qián cǎo*), *Rhizoma Pinelliae Praeparatu* *m (姜半夏 jiānɡ bàn xià)*, mulberry root bark (*桑白皮 sānɡ bái pí*), *Sophora flavescens (苦参 kǔ shēn)*, *Radix Paeoniae Alba (白芍 bái sháo)*, *Salvia miltiorrhiza (丹参 dān shēn)*, and *Ligusticum striatum (川芎 chuān xiōnɡ)* (Yan et al., 2020). Several clinical studies have demonstrated that Niaoduqing granules can reduce serum creatinine (SCr) in CKD patients and maintain a stable estimated glomerular filtration rate (eGFR) without serious adverse drug reactions (Zheng et al., 2017; Zheng et al., 2019).

However, most of these studies have only involved short-term observations (for approximately 1 year) and have focused on surrogate indicators, such as urine protein, hemoglobin (Hb), calcium (Ca^2+^), and phosphorous (P). Few of the studies on Niaoduqing granules have observed their efficacy on long-term prognoses, such as all-cause mortality and RRT occurrence. In addition, although CKD patients usually use Niaoduqing granules for a long time, given the long course of the disease, there remains a lack of evidence on Niaoduqing granules’ long-term efficacy. Hence, we collected clinical information via a retrospective cohort study to explore this.

## 2 Materials and methods

### 2.1 Study subjects

We searched the hospital information system (HIS) at Guangdong Provincial Hospital of Chinese Medicine (GPHCM) with the following keywords (in Chinese): “kidney disease” or “renal failure” or “renal disease” or “nephritis” or “proteinuria” or “hematuria.” Patients with kidney disease who visited from March 2012 to March 2023 were enrolled in a single-center, retrospective cohort study (ethics approval no. ZE 2023–330). Patients aged 18–80 years with CKD stages 3–5 (National Kidney Foundation, 2002) and who visited the nephrology department regularly (at least once every 6 months during the follow-up period) were eligible. Patients who had been diagnosed with acute and critical illnesses (e.g., acute cerebral infarction, acute heart failure, shock, malignant tumors, and hematological diseases) within 3 months of the baseline, had incomplete follow-up data, had received RRT (transplantation or dialysis) within 3 months from the baseline, had an eGFR less than 5 mL/min/1.73 m^2^, or had a follow-up period of less than 3 months were excluded.

Patients were grouped according to Niaoduqing granule prescription duration, defined as the time span from the first prescription date to the last prescription date during the follow-up duration. The prescription duration proportion was defined as prescription duration/follow-up duration. Participants with proportions over 80% were classified into the Niaoduqing granule (NDQ) group, and those with proportions of 0% were classified into the non-Niaoduqing granule (non-NDQ) group. Patients with proportions between 0% and 80% were excluded.

The composite outcome was defined as all-cause mortality, ESRD (received RRT or eGFR<5 mL/min/1.73 m^2^), and a ≥50% decline in the eGFR from baseline, along with at least a doubling of SCr. Patients were followed until the end of the follow-up time period (March 2023) or until they were lost to follow-up (defined as being unobservable during the time-at-risk period).

### 2.2 Ascertaining covariates

All covariates (except for sex) were time-updated and were collected at the baseline and once every 12 months over a 5-year follow-up duration. Demographic characteristics included age (continuous) and sex (fixed variables, male vs female). The clinical diagnosis included etiology, CKD stage, and comorbidities. The etiology included primary glomerulopathy, hypertensive nephropathy, diabetic nephropathy, other secondary kidney diseases (including infectious or autoimmune kidney diseases, obstructive nephropathy, uric acid nephropathy, and pyelonephritis), and unknown. Comorbidities included hypertension, diabetes, hyperuricemia, hyperlipidemia, anemia, cardiovascular diseases (CVDs), and cerebrovascular diseases.

Medication in use included ACEI/ARB, other antihypertensive drugs, hypoglycemic agents, urate-lowering drugs, lipid-lowering drugs, iron supplements, calcium supplements, sodium bicarbonate, ketoacid tablets, diuretics, and Chinese patent medicines. Chinese patent medicines were classified according to their clinical function in patients with CKD, such as removing turbidity, improving *qi* and *yin* deficiency (tonifying), and other functions.

Laboratory covariates included SCr, Hb, serum albumin (ALB), blood urea nitrogen (urea), total carbon dioxide (TCO_2_), uric acid (UA), P, Ca^2+^, potassium (K^+^), sodium (Na^+^), low-density lipoprotein cholesterol (LDL-C), total cholesterol (TC), high-density lipoprotein cholesterol (HDL-C), fasting blood glucose (Glu), and the protein/creatinine ratio (PCR). The eGFR was calculated using the CKD Epidemiology Collaboration (CKD-EPI) creatinine equation (KDIGO, 2021a).

## 3 Statistical analyses

Continuous variables were summarized using the mean ± standard deviation (SD) for normal distributions and median (interquartile ranges) for non-normal distributions. Categorical variables were summarized using frequencies and percentages. The Mann–Whitney *U*-test and chi-square test were applied to compare between-group characteristics.

For time-updated covariates, line graphs were plotted to visualize annual variations and estimated marginal SCr means. After standardizing all the time-updated continuous variables and transforming the follow-up duration to five consecutive visits, a generalized linear mixed model (GLMM) was constructed with individual random effects and group fixed effects.

For survival analysis, covariates with a missing rate greater than 40% were excluded, and outliers of continuous variables were winsorized with 0.1 cut-offs at each tail. Missing data were imputed using a random forest. A 1:2 nearest neighbor propensity score matching (PSM) with replacement within a caliper of 0 was applied to achieve covariate balance. The survival probability was compared using the Kaplan–Meier curve and a log-rank test. Variables with *p* < 0.10 in univariate Cox regression were included in multivariate Cox regression with a “backward” stepwise method. The Schoenfeld residual test was performed to check the proportional hazards assumption. The hazard ratio (HR) and 95% confidence interval (95% CI) values were used to describe the results. A subgroup analysis with interaction effects was conducted by stratification of age, sex, PCR, CKD stage, with or without hypertension, diabetes, and etiology. A forest plot was used to show the results.

All statistical tests were two-sided, and *p*-values <0.05 were considered statistically significant. All statistical analyses were conducted via R Studio 4.3.0 and SPSS 25.0.

## 4 Results

### 4.1 Cohort characteristics

**FIGURE 1:**
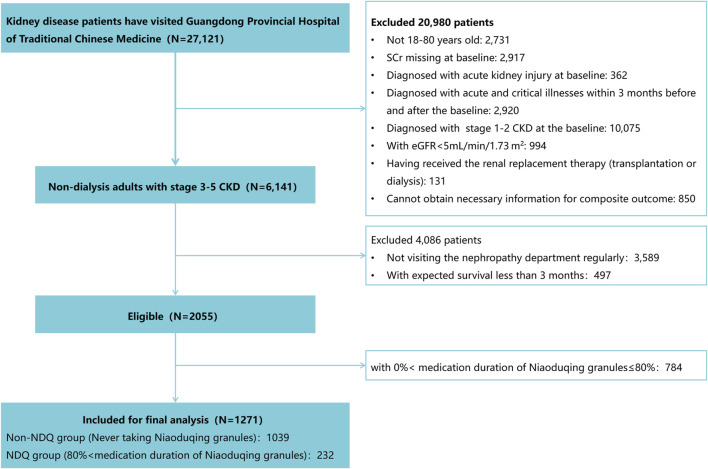
Recruitment flow chart.

Of the 27,121 patients with kidney disease, 2,731 who were not non-dialysis adults with CKD stages 3–5 with complete information for composite outcome and 4,086 who were not regularly visiting a nephrologist or had an expected survival duration of less than 3 months were excluded. Moreover, 784 patients did not meet the 0%–80% prescription duration requirement. Ultimately, 1,271 participants were included in the final analysis, with 232 in the NDQ group and 1,039 in the non-NDQ group (Figure 1).

The 1,271 participants had a median age of 58.30 (46.31, 66.97) years, and 585 (46.03%) were females. A total of 777 (61.13%) of the individuals had CKD stage 3, 273 (21.48%) had CKD stage 4, and 221 (17.39%) had CKD stage 5. The endpoint occurrence was 356 (28.01%), with a median follow-up duration of 29.71 (12.10, 56.07) months. The NDQ group had an endpoint occurrence of 84 (36.21%), with a median follow-up duration of 44.38 (23.27, 68.93) months. The non-NDQ group had an endpoint occurrence of 272 (26.18%), with a median follow-up duration of 26.85 (11.21, 52.46) months. Some covariates could not reach inter-group balance. These were CKD stage, Hb, urea, TCO_2_, HDL-C, etiology, CVDs, ACEI/ARB, other antihypertensive drugs, iron supplements, sodium bicarbonate, ketoacid tablets, and Chinese patent medicines (*p* < 0.05) (important laboratory data are highlighted in Table 1, and details can be found in Supplementary Table S1).

**TABLE 1:** Important laboratory characteristics before and after propensity score matching.

	Unmatched			*P*	Matched			*p*
	Overall (N = 1,271)	Non-NDQ (N = 1,039)	NDQ (N = 232)		Overall (N = 563)	Non-NDQ (N = 331)	NDQ (N = 232)	
Hb, g/L	120.72 (104.00, 133.21)	121.00 (105.00, 134.00)	117.00 (102.00, 132.00)	0.039	115.00 (101.00, 131.00)	114.00 (100.00, 130.00)	117.00 (102.00, 132.00)	0.475
ALB, g/L	42.20 (38.60, 44.77)	42.20 (38.70, 44.80)	41.92 (38.28, 44.41)	0.513	41.90 (38.05, 44.35)	41.90 (37.70, 44.30)	41.92 (38.28, 44.41)	0.528
UA, mmol/L	444.00 (384.00, 512.00)	442.00 (383.50, 511.00)	454.50 (387.75, 522.00)	0.143	451.00 (384.00, 516.00)	449.00 (381.50, 513.62)	454.50 (387.75, 522.00)	0.407
Urea, mmol/L	9.51 (7.16, 14.16)	9.10 (7.04, 13.88)	11.08 (8.11, 14.68)	0.000	10.83 (7.95, 15.10)	10.64 (7.89, 15.84)	11.08 (8.11, 14.68)	0.745
TCO_2_, mmol/L	23.50 (21.25, 25.40)	23.60 (21.45, 25.50)	22.90 (20.90, 24.63)	0.001	23.00 (20.70, 24.80)	23.00 (20.50, 24.95)	22.90 (20.90, 24.63)	0.556
P, mmol/L	1.25 (1.15, 1.40)	1.25 (1.15, 1.39)	1.28 (1.15, 1.42)	0.194	1.27 (1.16, 1.43)	1.27 (1.17, 1.44)	1.28 (1.15, 1.42)	0.561
Ca^2+^, mmol/L	2.29 (2.21, 2.37)	2.29 (2.21, 2.37)	2.30 (2.22, 2.36)	0.764	2.29 (2.20, 2.36)	2.28 (2.19, 2.36)	2.30 (2.22, 2.36)	0.274
K^+^, mmol/L	4.41 (4.21, 4.70)	4.40 (4.21, 4.69)	4.45 (4.22, 4.76)	0.092	4.46 (4.23, 4.76)	4.46 (4.23, 4.76)	4.45 (4.22, 4.76)	0.993
Na^+^, mmol/L	140.89 (140.00, 141.54)	140.89 (140.00, 141.51)	140.92 (140.00, 142.00)	0.525	140.87 (139.97, 141.85)	140.82 (139.92, 141.49)	140.92 (140.00, 142.00)	0.405
LDL-C, mmol/L	3.25 (2.72, 3.88)	3.28 (2.75, 3.90)	3.16 (2.60, 3.80)	0.077	3.22 (2.67, 3.94)	3.27 (2.75, 4.04)	3.16 (2.60, 3.80)	0.104
TC, mmol/L	5.03 (4.40, 5.75)	5.08 (4.43, 5.75)	4.95 (4.25, 5.73)	0.064	4.98 (4.36, 5.81)	5.01 (4.45, 5.86)	4.95 (4.25, 5.73)	0.107
HDL-C, mmol/L	1.26 (1.10, 1.44)	1.27 (1.11, 1.45)	1.21 (1.03, 1.43)	0.019	1.23 (1.04, 1.42)	1.24 (1.05, 1.41)	1.21 (1.03, 1.43)	0.488
Glu, mmol/L	5.56 (5.28, 6.23)	5.56 (5.30, 6.36)	5.52 (5.17, 5.97)	0.067	5.54 (5.27, 6.27)	5.56 (5.31, 6.50)	5.52 (5.17, 5.97)	0.072
PCR, mg/g	1.28 (0.57, 2.45)	1.28 (0.57, 2.47)	1.30 (0.54, 2.39)	0.819	1.38 (0.65, 2.58)	1.46 (0.71, 2.74)	1.30 (0.54, 2.39)	0.060

Note: hemoglobin, Hb; albumin, ALB; uric acid, UA; blood urea nitrogen, urea; total carbon dioxide, TCO_2_; phosphorous, P; calcium, Ca^2+^; potassium, K^+^; sodium, Na^+^; low-density lipoprotein cholesterol, LDL-C; total cholesterol, TC; high-density lipoprotein cholesterol, HDL-C; fasting blood glucose, Glu; protein/creatinine ratio, PCR.

### 4.2 Visualizing SCr variation

**FIGURE 2:**
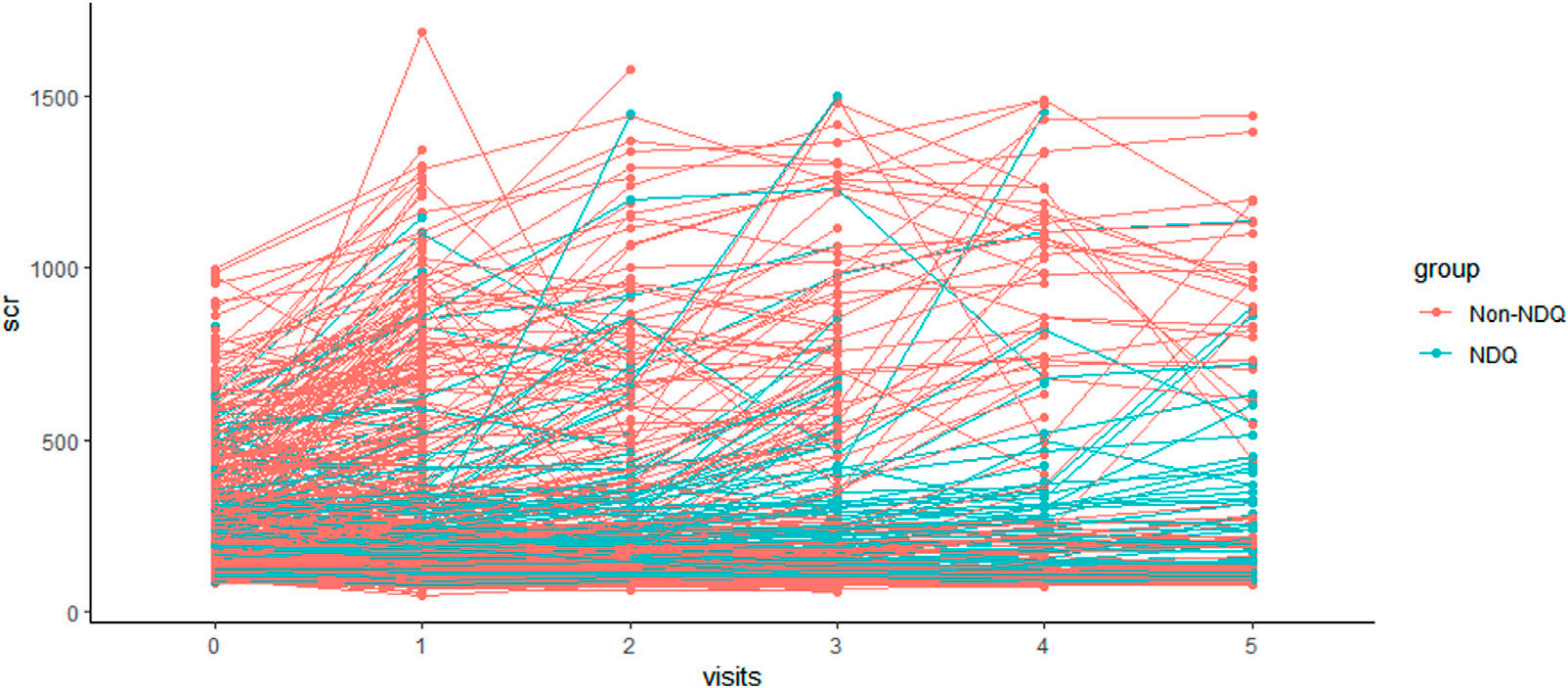
SCr variation of each individual.

**FIGURE 3:**
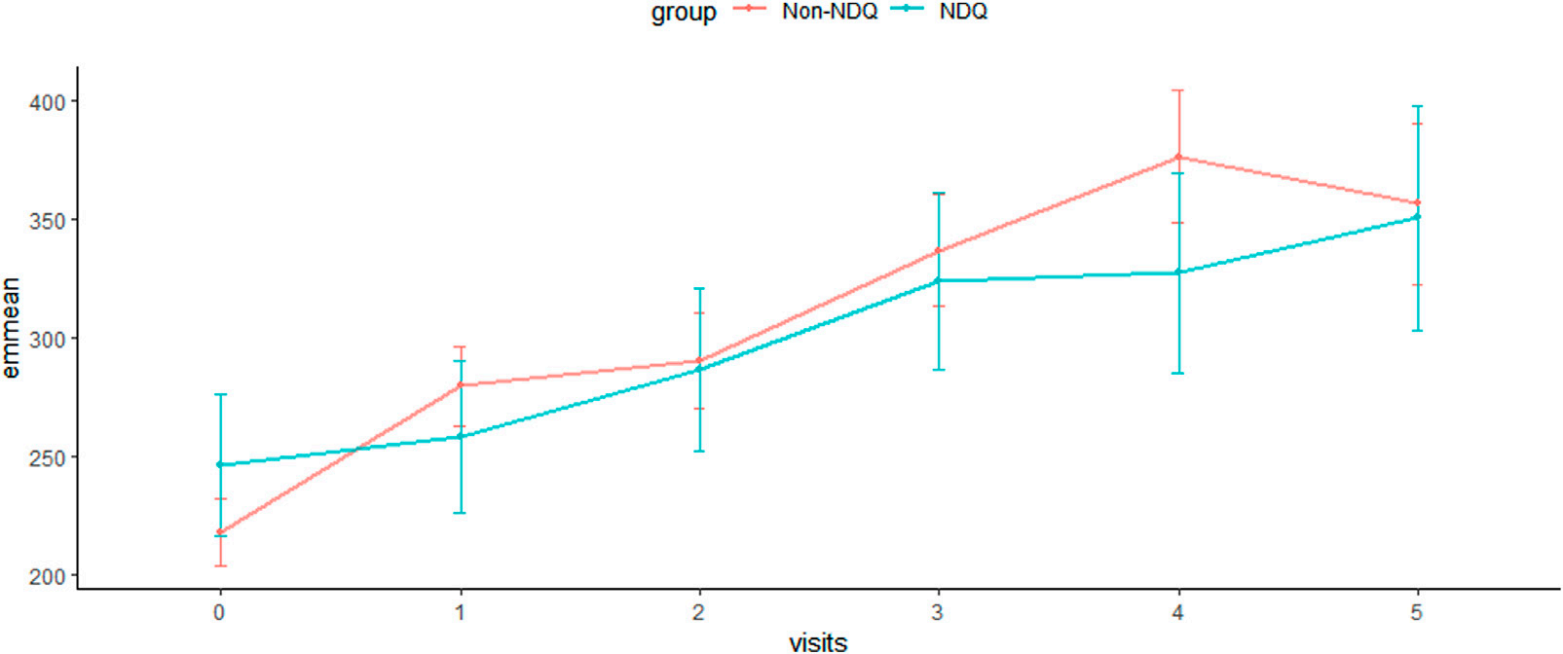
Estimated marginal means of SCr.

Line graphs indicated that SCr steadily increased over time in both NDQ and non-NDQ groups (Figure 2 and Figure 3). The NDQ group showed a higher SCr level than the non-NDQ group at the baseline, and it had a lower estimated marginal SCr mean than the non-NDQ group during the follow-up period. Additionally, the NDQ group had a smaller SCr slope than the non-NDQ group.

### 4.3 Generalized linear mixed model

A generalized linear mixed model was constructed after adjusting the fixed and time-updated covariates. It had an F-statistic of 41.333, an Akaike information criterion (AIC) of 1,980.974, and a Bayesian information criterion (BIC) of 2,001.298 (*p* < 0.001). Among the 1,271 participants, the coefficients from visit 1 to visit 5 were 0.134 (95% CI 0.060, 0.208), 0.266 (95% CI 0.165, 0.368), 0.420 (95% CI 0.292, 0.547), 0.507 (95% CI 0.343, 0.671), and 0.347 (95% CI 0.140, 0.554) in overall participants compared to the baseline (*p* < 0.05), respectively. This indicated that SCr levels trended upward from visit 1 to visit 5, which was consistent (Figure 2 and Figure 3).

The mean SCr Z-scores for the non-NDQ and NDQ groups were −0.175 and 0.153, respectively, at baseline, with *p* = 0.015. This indicated significantly higher SCr levels in the NDQ group than in the non-NDQ group. Compared to the non-NDQ group, the NDQ group’s coefficients (95% CI) from visit 1 to visit 5 were 0.207 (95% CI: 0.346, −0.068), −0.214 (95% CI: 0.389, −0.039), −0.324 (95% CI: 0.538, −0.109), −0.502 (95% CI: 0.761, −0.243), and −0.252 (95% CI: 0.569, 0.065), with a significant *p-*value from visit 1 to visit 4. This indicated that the NDQ group’s mean SCr was significantly lower than that of the non-NDQ group from visit 1 to visit 4. This was also the case for visit 5; however, the results were not statistically significant (Table 2).

**TABLE 2:** Fixed coefficients from the generalized linear mixed model.

		Coefficient	95% CI	Standard error	*t*	*p*
	Intercept	−0.175	(-0.345, 0.006)	0.087	−2.026	0.043
Overall comparison						
	Baseline	Reference				
	Visit 1	0.134	(0.060, 0.208)	0.038	3.544	0.000
	Visit 2	0.266	(0.165, 0.368)	0.052	5.142	0.000
	Visit 3	0.420	(0.292, 0.547)	0.065	6.458	0.000
	Visit 4	0.507	(0.343, 0.671)	0.084	6.066	0.000
	Visit 5	0.347	(0.140, 0.554)	0.105	3.294	0.001
Group comparison						
Baseline	Non-NDQ	Reference				
	NDQ	0.153	(0.030, 0.276)	0.063	2.443	0.015
Visit 1	Non-NDQ	Reference				
	NDQ	−0.207	(-0.346, −0.068)	0.071	−2.921	0.004
Visit 2	Non-NDQ	Reference				
	NDQ	−0.214	(-0.389, −0.039)	0.089	−2.397	0.017
Visit 3	Non-NDQ	Reference				
	NDQ	−0.324	(-0.538, −0.109)	0.109	−2.959	0.003
Visit 4	Non-NDQ	Reference				
	NDQ	−0.502	(-0.761, −0.243)	0.132	−3.804	0.000
Visit 5	Non-NDQ	Reference				
	NDQ	−0.252	(-0.569, 0.065)	0.162	−1.561	0.119

### 4.4 Survival analysis

#### 4.4.1 Comparison of survival probability

**FIGURE 4:**
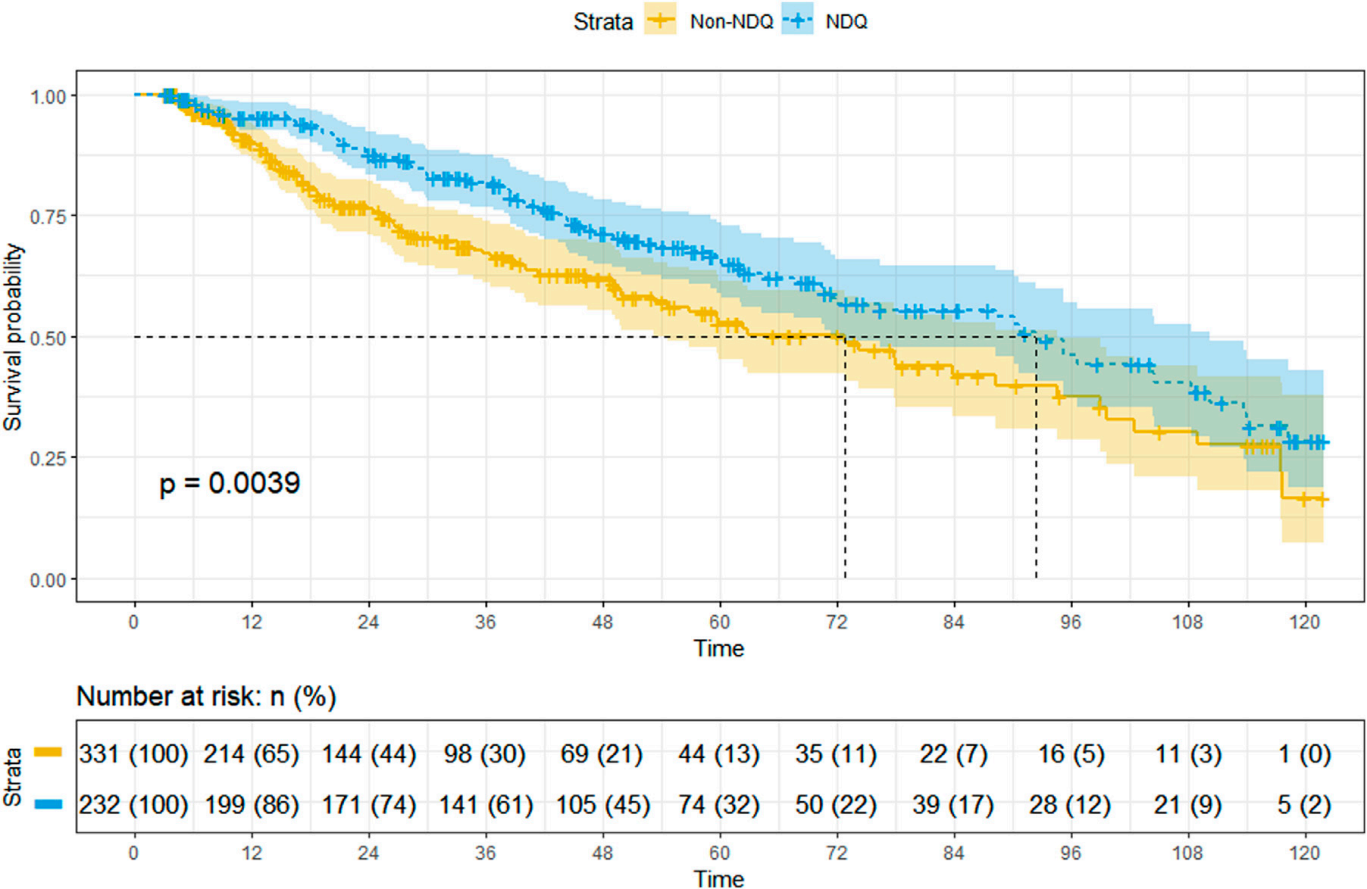
Kaplan-Meier curve.

A total of 563 patients were included for survival analysis, with 188 (33.39%) reaching the endpoint and a median survival duration of 77.9 (67.6, 95.2) months. Of the included patients, 232 were from the NDQ group, with a composite outcome of 84 (36.21%) and a median survival duration of 92.4 (71.7, 110.2) months, and 331 were from the non-NDQ group, with a composite outcome of 104 (31.42%) and a median survival duration of 72.7 (54.7, 94.6) months. The Kaplan–Meier curves showed that the NDQ group had a higher survival probability than the non-NDQ group (*p* = 0.0039) (Figure 4).

#### 4.4.2 Cox regression

A total of 24 variables were significant in univariate Cox regression with *p* < 0.10: NDQ group, age, CKD stage, Hb, ALB, urea, TCO_2_, P, Ca^2+^, K^+^, PCR, hypertension, diabetes, hyperuricemia, anemia, other antihypertensive drugs, hypoglycemic drugs, lipid-lowering drugs, calcium supplements, iron supplements, sodium bicarbonate, ketoacid tablets, diuretics, and turbidity-removing Chinese patent medicines (Supplementary Table S2).

As presented in Table 3, three models were constructed: model 1 was a univariate Cox model of exposure; model 2 further adjusted the significant comorbidities based on model 1; and model 3 further adjusted other significant covariates in univariate Cox regression based on model 2. The HR of the NDQ group in models 1 and 2 was 0.654 (95% CI: 0.489, 0.875, *p* = 0.004) and 0.646 (95% CI: 0.476, 0.877, *p* = 0.005), respectively. After adjusting for all covariates, the NDQ group still had a significant HR (95% CI) in model 3 (HR: 0.602, 95% CI: 0.441, 0.820, *p* = 0.001). These results indicated that taking Niaoduqing granules is an independent protective factor for thwarting long-term decline among CKD patients at stages 3–5.

**TABLE 3:** Cox regression results.

		HR (95% CI)	*P*
Model 1			
Group	Non-NDQ	Reference	
	NDQ	0.654 (0.489, 0.875)	0.004
Model 2			
Group	Non-NDQ	Reference	
	NDQ	0.646 (0.476, 0.877)	0.005
Age		0.977 (0.966, 0.988)	0.000
CKD stage	Stage 3	Reference	
	Stage 4	2.034 (1.368, 3.024)	0.000
	Stage 5	2.193 (1.223, 3.931)	0.008
Hb		0.980 (0.971, 0.988)	0.000
Urea		1.046 (1.010, 1.084)	0.011
PCR		1.094 (1.035, 1.156)	0.001
Hypertension	No	Reference	
	Yes	1.632 (1.148, 2.319)	0.006
Diabetes	No	Reference	
	Yes	1.467 (1.016, 2.117)	0.041
Model 3			
Group	Non-NDQ	Reference	
	NDQ	0.602 (0.442, 0.820)	0.001
Age		0.978 (0.967, 0.989)	0.000
CKD stage	Stage 3		
	Stage 4	2.102 (1.415, 3,122)	0.000
	Stage 5	2.006 (1.114, 3.615)	0.020
Hb		0.979 (0.970, 0.987)	0.000
Urea		1.057 (1.021, 1.095)	0.002
PCR		1.131 (1.070, 1.194)	0.000
Hypertension	No	Reference	
	Yes	1.591 (1.120, 2.259)	0.009
Hypoglycemic drug	No	Reference	
	Yes	1.636 (1.146, 2.335)	0.007
Calcium supplement	No	Reference	
	Yes	0.638 (0.443, 0.918)	0.015
Sodium bicarbonate	No	Reference	
	Yes	1.308 (0.950, 1.800)	0.100

Note: model 1 was a univariate group model; model 2 was further adjusted for age, CKD stage, Hb; ALB, urea, TCO_2_, P, Ca^2+^, K^+^, PCR, hypertension, diabetes, hyperuricemia, and anemia; and model 3 was further adjusted for other antihypertensive drugs, hypoglycemic drugs, lipid-lowering drugs, calcium supplements, iron supplements, sodium bicarbonate, ketoacid tablets, diuretics, turbidity-removing Chinese patent medicines, and tonifying Chinese patent medicines. The models were constructed with a “backward” stepwise regression.

#### 4.4.3 Subgroup analyses

**FIGURE 5:**
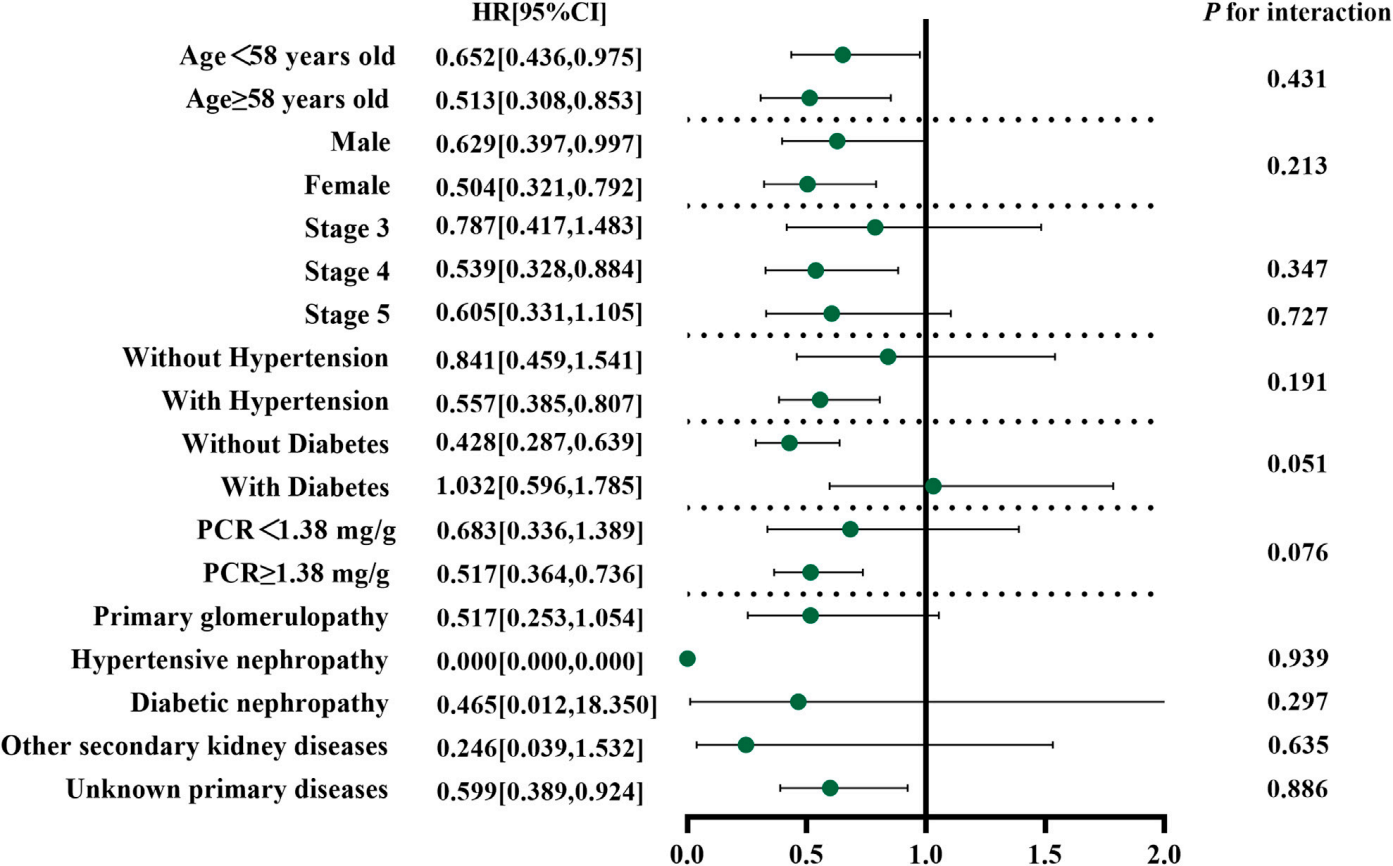
Forest plot of subgroup analysis.

A subgroup analysis was stratified by median age, sex, CKD stage, median PCR, hypertension, diabetes, and etiology based on model 3. The findings showed no interaction effect in the subgroup analysis (*p >* 0.05) (Figure 5).

## 5 Discussion

Our retrospective cohort study showed the long-term efficacy of Niaoduqing granules on non-dialysis CKD patients at stages 3–5. The results of the GLMM and survival analysis indicated that the participants taking Niaoduqing granules over the long term had a more steady growth in their SCr levels and a higher survival probability. Taking Niaoduqing granules was an independent protective factor for thwarting CKD progression (HR: 0.602, 95% CI: 0.441, 0.820, *p* = 0.001). Without any stratification, there was an interaction effect on the correlation between the NDQ group and the composite outcome.

CKD at stages 3–5 is known as “consumptive disease” or “edema” in traditional Chinese medicine. The pathogenesis involves spleen and kidney deficiency and retention of dampness, turbidity, and stasis, which lead to toxins congesting the body and can cause a series of diseases. In Niaoduqing granules, *Radix Astragali (黄芪 huánɡ qí)* and *Codonopsis pilosula (党参 dǎnɡ shēn)* can invigorate the spleen and supplement *qi*; *Radix Polygoni Multiflori Preparata (制何首乌 zhì hé shǒu wū)* nourishes the kidneys and strengthens essence; *Radix et Rhizoma Rhei (生大黄 shēnɡ dà huánɡ)* and *Radix Paeoniae Alba (白芍 bái sháo)* clear Fu organs and expel turbidity; *Rhizoma A. macrocephalae (白术 bái zhú)* strengthens the spleen and removes dampness; *Wolfiporia extensa (茯苓 fú línɡ)* and *Plantago asiatica (车前草 chē qián cǎo)* remove dampness; *Rhizoma Pinelliae Praeparatum (姜半夏 jiānɡ bàn xià)*, mulberry root bark (*桑白皮 sānɡ bái pí*), and *S. flavescens (苦参 kǔ shēn)* eliminate dampness and harmonize the center; and *L. striatum (川芎 chuān xiōnɡ)* and *S. miltiorrhiza (丹参 dān shēn)* activate blood circulation and remove stasis. These ingredients work synergistically to strengthen the spleen and the kidneys, clear Fu organs, expel turbidity, activate blood circulation, and dissolve stasis. Theoretically, the effects of Niaoduqing granules should be consistent with the pathogenesis of CKD stages 3–5, promoting the expulsion of turbid toxins and thwarting disease progression.

Research has shown that the bioactive components of Niaoduqing granules might regulate M2 microglia and mediate the signal transducer and activator of transcription 6 (STAT6) and phosphoinositide-3 kinase γ (PI3Kγ) signaling pathways by affecting the phosphatase and tensin homolog (PTEN) and Jumonji domain-containing protein-3 (JMJD3) (Liu et al., 2020; Jiao et al., 2021a; Jiao et al., 2021b; An et al., 2022; Li et al., 2022; An et al., 2023; Liu et al., 2023). This can suppress tubular epithelial–mesenchymal cell transformation, improve microinflammatory states, reduce oxidative stress, and alleviate insulin resistance to mitigate renal fibrosis (Wei, 2013; Wang et al., 2017; Ai et al., 2021; Xu et al., 2023). Furthermore, Niaoduqing granules affect several amino acid metabolism pathways by regulating the intestinal flora to improve kidney function (Wu et al., 2016). Thus, its pharmacological and metabolomic mechanisms might relate to various bioactive ingredients, such as astragaloside, emodin, isoflavonoids, salvianolic acid A, and paeoniflorin (Kong et al., 2021; Zhao and Li, 2022). These ingredients may serve as an explanation for the clinical efficacy of Niaoduqing granules in modern pharmacology.

Our results were consistent with several clinical studies (Zheng et al., 2019; Song et al., 2022; Zhong et al., 2022) and offered several additional advantages. First, to the best of our knowledge, our study utilized the largest sample size of any study on Niaoduqing granules to date. We collected as many clinical characteristics as possible to imitate a real-world setting. It provided valuable data for supporting the long-term efficacy and safety of Niaoduqing granules. Second, the study used GLMM, which combined fixed and random effects to maximize the interpretation of data variability. PSM was used to reduce selection bias, and COX regression models were used to analyze the effect of independent variables on survival time and composite outcomes to minimize the effects of any confounding factors. Through these analyses, it was possible to demonstrate the clinical efficacy of Niaoduqing granules in the composite outcomes of CKD progression.

Our study also has several limitations. First, we did not collect or analyze the characteristics of TCM syndrome differentiation or the symptoms due to the limitations of a retrospective study. We also did not analyze the characteristics of Chinese herbal decoctions because most participants regularly took Chinese herbal decoctions at GPHCM. Second, the prescription duration did not cover any diagnosis or medication records from other medical institutions, and medication adherence was not assessed during the follow-up period. This may have introduced a selection bias. Third, although we adjusted for unbalanced covariates in model 2, there may still have been confounding effects. Fourth, although our study utilized the largest sample size, prospective studies with larger sample sizes will be necessary for further research.

## Data Availability

The raw data supporting the conclusion of this article will be made available by the authors, without undue reservation.

## Abbreviations

AIC, Akaike information criterion; ACEIs, angiotensin-converting enzyme inhibitors; ARBs, angiotensin receptor blockers; ALB, serum albumin; BIC, Bayesian information criterion; CKD, chronic kidney disease; Ca^2+^, calcium; CVDs, cardiovascular diseases; eGFR, estimated glomerular filtration rate; ESRD, end-stage kidney disease; GPHCM, Guangdong Provincial Hospital of Chinese Medicine; GFR, glomerular filtration rate; Glu, fasting blood glucose; GLMM, generalized linear mixed model; HIS, hospital information system; Hb, hemoglobin; HR, hazard ratio; HDL-C, high-density lipoprotein cholesterol; K^+^, potassium; LDL-C, low-density lipoprotein cholesterol; NDQ, Niaoduqing group; Na^+^, sodium; 95% CI, 95% confident interval; P, phosphorous; PCR, protein/creatinine ratio; PSM, propensity score matching; RRT, renal replacement therapy; SCr, serum creatinine; SGLT2i, sodium–glucose cotransporter-2 inhibitors; SE, standard error; TCO_2_, total carbon dioxide; TC, total cholesterol; urea, blood urea nitrogen; UA, uric acid.
