# Supplementary material for: A Chinese patent medicine’s long-term efficacy on non-dialysis patients with CKD stages 3–5: a retrospective cohort study
Source: Front Pharmacol. 2024 Apr 26;15:1379338. doi: 10.3389/fphar.2024.1379338 (PMC11082339; doi:10.3389/fphar.2024.1379338)
Supplement: Supplementary file 1 [file Table1.docx]

**Supplementary Table 1 Baseline characteristics before and after propensity score matching**

|  | Unmatched | | |  | Matched | | |  |
| --- | --- | --- | --- | --- | --- | --- | --- | --- |
|  | Overall (N=1,271) | Non-NDQ  (N=1,039) | NDQ  (N=232) | P | Overall (N=563) | Non-NDQ  (N=331) | NDQ (N=232) | P |
| Age | 58.30 (46.31, 66.97) | 58.13 (45.80, 66.84) | 59.06 (47.82, 67.80) | 0.327 | 57.92 (46.13, 66.53) | 56.72 (44.80, 65.63) | 59.06 (47.82, 67.80) | 0.081 |
| Sex (%) | | | | | | | | |
| Male | 686 (53.97) | 558 (53.71) | 128 (55.17) | 0.740 | 324 (57.55) | 196 (59.21) | 128 (55.17) | 0.385 |
| Female | 585 (46.03) | 481 (46.29) | 104 (44.83) |  | 239 (42.45) | 135 (40.79) | 104 (44.83) |  |
| CKD stage (%) |  |  |  |  |  |  |  |  |
| Stage 3 | 777 (61.13) | 676 (65.06) | 101 (43.53) | <0.001 | 265 (47.07) | 164 (49.55) | 101 (43.53) | 0.365 |
| Stage 4 | 273 (21.48) | 190(18.29) | 83 (35.78) |  | 187 (33.21) | 104 (31.42) | 83 (35.78) |  |
| Stage 5 | 221 (17.39) | 173 (16.65) | 48 (20.69) |  | 111 (19.72) | 63 (19.03) | 48 (20.69) |  |
| Follow-up duration | 29.71 (12.10, 56.07) | 26.85 (11.21, 52.46) | 44.38 (23.27, 68.93) | <0.001 | 28.46 (11.21, 54.43) | 19.05 (8.93, 43.05) | 44.38 (23.27, 68.93) | <0.001 |
| Composite endpoint (%) | | | | | | | | |
| No | 915 (71.99) | 767 (73.82) | 148 (63.79) | 0.003 | 375 (66.61) | 227 (68.58) | 148 (63.79) | 0.274 |
| Yes | 356 (28.01) | 272 (26.18) | 84 (36.21) |  | 188 (33.39) | 104 (31.42) | 84 (36.21) |  |
| Medication | | | | | | | | |
| Etiology (%) | | | | | | | | |
| Primary Glomerulopathy | 389 (30.61) | 343 (33.01) | 46 (19.83) | <0.001 | 147 (26.11) | 101 (30.51) | 46 (19.83) | 0.026 |
| Hypertensive nephropathy | 28 (2.20) | 18 (1.73) | 10 (4.31) |  | 19 (3.37) | 9 (2.72) | 10 (4.31) |  |
| Diabetic nephropathy | 67 (5.27) | 53 (5.10) | 14 (6.03) |  | 28 (4.97) | 14 (4.23) | 14 (6.03) |  |
| Other secondary kidney disease | 97 (7.63) | 68 (6.54) | 29 (12.50) |  | 55 (9.77) | 26 (7.85) | 29 (12.50) |  |
| Unknown primary disease | 690 (54.29) | 557 (53.61) | 133 (57.33) |  | 314 (55.77) | 181 (54.68) | 133 (57.33) |  |
| Hypertension (%) | | | |  |  |  |  |  |
| No | 504 (39.65) | 429 (41.29) | 75 (32.33) | 0.014 | 199 (35.35) | 124 (37.46) | 75 (32.33) | 0.244 |
| Yes | 767 (60.35) | 610 (58.71) | 157 (67.67) |  | 364 (64.65) | 207 (62.54) | 157 (67.67) |  |
| Diabetes (%) | | | |  |  |  |  |  |
| No | 938 (73.80) | 765 (73.63) | 173 (74.57) | 0.832 | 404 (71.76) | 231 (69.79) | 173 (74.57) | 0.252 |
| Yes | 333 (26.20) | 274 (26.37) | 59 (25.43) |  | 159 (28.24) | 100 (30.21) | 59 (25.43) |  |
| Hyperlipidemia (%) | | | |  |  |  |  |  |
| No | 993 (78.13) | 815 (78.44) | 178 (76.72) | 0.628 | 440 (78.15) | 262 (79.15) | 178 (76.72) | 0.560 |
| Yes | 278 (21.87) | 224 (21.56) | 54 (23.28) |  | 123 (21.85) | 69 (20.85) | 54 (23.28) |  |
| Hyperuricemia (%) | | | |  |  |  |  |  |
| No | 823 (64.75) | 678 (65.26) | 145 (62.50) | 0.473 | 365 (64.83) | 220 (66.47) | 145 (62.50) | 0.379 |
| Yes | 448 (35.25) | 361 (34.74) | 87 (37.50) |  | 198 (35.17) | 111 (33.53) | 87 (37.50) |  |
| Anemia (%) | | | |  |  |  |  |  |
| No | 1,122 (88.28) | 916 (88.16) | 206 (88.79) | 0.875 | 488 (86.68) | 282 (85.20) | 206 (88.79) | 0.267 |
| Yes | 149 (11.72) | 123 (11.84) | 26 (11.21) |  | 75 (13.32) | 49 (14.80) | 26 (11.21) |  |
| CVDs (%) | | | |  |  |  |  |  |
| No | 1,113 (87.57) | 920 (88.55) | 193 (83.19) | 0.034 | 488 (86.68) | 295 (89.12) | 193 (83.19) | 0.056 |
| Yes | 158 (12.43) | 119 (11.45) | 39 (16.81) |  | 75 (13.32) | 36 (10.88) | 39 (16.81) |  |
| Cerebrovascular disease (%) | | | |  |  |  |  |  |
| No | 1,145 (90.09) | 933 (89.80) | 212 (91.38) | 0.544 | 513 (91.12) | 301 (90.94) | 212 (91.38) | 0.975 |
| Yes | 126 (9.91) | 106 (10.20) | 20 (8.62) |  | 50 (8.88) | 30 (9.06) | 20 (8.62) |  |
| ACEI/ARB (%) | | | |  |  |  |  |  |
| No | 823 (64.75) | 655 (63.04) | 168 (72.41) | 0.009 | 394 (69.98) | 226 (68.28) | 168 (72.41) | 0.337 |
| Yes | 448 (35.25) | 384 (36.96) | 64 (27.59) |  | 169 (30.02) | 105 (31.72) | 64 (27.59) |  |
| Other antihypertensive drugs (%) | | | |  |  |  |  |  |
| No | 790 (62.16) | 675 (64.97) | 115 (49.57) | <0.001 | 329 (58.44) | 214 (64.65) | 115 (49.57) | <0.001 |
| Yes | 481 (37.84) | 364 (35.03) | 117 (50.43) |  | 234 (41.56) | 117 (35.35) | 117 (50.43) |  |
| Hypoglycemic agents (%) | | | |  |  |  |  |  |
| No | 1,019 (80.17) | 840 (80.85) | 179 (77.16) | 0.236 | 446 (79.22) | 267 (80.66) | 179 (77.16) | 0.366 |
| Yes | 252 (19.83) | 199 (19.15) | 53 (22.84) |  | 117 (20.78) | 64 (19.34) | 53 (22.84) |  |
| Urate-lowering drugs (%) | | | |  |  |  |  |  |
| No | 966 (76.00) | 800 (77.00) | 166 (71.55) | 0.095 | 406 (72.11) | 240 (72.51) | 166 (71.55) | 0.878 |
| Yes | 305 (24.00) | 239 (23.00) | 66 (28.45) |  | 157 (27.89) | 91 (27.49) | 66 (28.45) |  |
| Lipid-lowering drugs (%) | | | |  |  |  |  |  |
| No | 910 (71.60) | 745 (71.70) | 165 (71.12) | 0.922 | 400 (71.05) | 235 (71.00) | 165 (71.12) | 1.000 |
| Yes | 361 (28.40) | 294 (28.30) | 67 (28.88) |  | 163 (28.95) | 96 (29.00) | 67 (28.88) |  |
| Calcium supplements (%) | | | |  |  |  |  |  |
| No | 1,000 (78.68) | 813 (78.25) | 187 (80.60) | 0.482 | 443 (78.69) | 256 (77.34) | 187 (80.60) | 0.409 |
| Yes | 271 (21.32) | 226 (21.75) | 45 (19.40) |  | 120 (21.31) | 75 (22.66) | 45 (19.40) |  |
| Iron supplements (%) | | | |  |  |  |  |  |
| No | 986 (77.58) | 825 (79.40) | 161 (69.40) | 0.001 | 411 (73.00) | 250 (75.53) | 161 (69.40) | 0.129 |
| Yes | 285 (22.42) | 214 (20.60) | 71 (30.60) |  | 152 (27.00) | 81 (24.47) | 71 (30.60) |  |
| Sodium bicarbonate (%) | | | |  |  |  |  |  |
| No | 776 (61.05) | 691 (66.51) | 85 (36.64) | <0.001 | 282 (50.09) | 197 (59.52) | 85 (36.64) | <0.001 |
| Yes | 495 (38.95) | 348 (33.49) | 147 (63.36) |  | 281 (49.91) | 134 (40.48) | 147 (63.36) |  |
| Ketoacid tablets (%) | | | |  |  |  |  |  |
| No | 911 (71.68) | 783 (75.36) | 128 (55.17) | <0.001 | 363 (64.48) | 235 (71.00) | 128 (55.17) | <0.001 |
| Yes | 360 (28.32) | 256 (24.64) | 104 (44.83) |  | 200 (35.52) | 96 (29.00) | 104 (44.83) |  |
| Diuretics (%) | | | |  |  |  |  |  |
| No | 1,054 (82.93) | 867 (83.45) | 187 (80.60) | 0.345 | 461 (81.88) | 274 (82.78) | 187 (80.60) | 0.583 |
| Yes | 217 (17.07) | 172 (16.55) | 45 (19.40) |  | 102 (18.12) | 57 (17.22) | 45 (19.40) |  |
| Turbidity-removing Chinese patent medicines (%) | | | |  |  |  |  |  |
| No | 868 (68.29) | 796 (76.61) | 72 (31.03) | <0.001 | 318 (56.48) | 246 (74.32) | 72 (31.03) | <0.001 |
| Yes | 403 (31.71) | 243 (23.39) | 160 (68.97) |  | 245 (43.52) | 85 (25.68) | 160 (68.97) |  |
| Tonifying Chinese patent medicines (%) | | | |  |  |  |  |  |
| No | 1,040 (81.83) | 864 (83.16) | 176 (75.86) | 0.012 | 463 (82.24) | 287 (86.71) | 176 (75.86) | 0.001 |
| Yes | 231 (18.17) | 175 (16.84) | 56 (24.14) |  | 100 (17.76) | 44 (13.29) | 56 (24.14) |  |
| Other types of Chinese patent medicines (%) | | | |  |  |  |  |  |
| No | 1,088 (85.60) | 881 (84.79) | 207 (89.22) | 0.102 | 498 (88.45) | 291 (87.92) | 207 (89.22) | 0.731 |
| Yes | 183 (14.40) | 158 (15.21) | 25 (10.78) |  | 65 (11.55) | 40 (12.08) | 25 (10.78) |  |

Note:Primary Glomerulonephritides included chronic nephritis, nephropathy syndrome and IgA nephropathy.Other secondary nephrosis included systemic lupus erythematosus nephritis, Henoch-Schonlein purpura,Hepatitis B virus-associated nephritis and obstructive nephropathy, etc.;Cardiovascular diseases, CVDs; Angiotensin-converting enzyme inhibitors, ACEIs; Angiotensin receptor blockers, ARBs.
