# Supplementary material for: A Chinese patent medicine’s long-term efficacy on non-dialysis patients with CKD stages 3–5: a retrospective cohort study
Source: Front Pharmacol. 2024 Apr 26;15:1379338. doi: 10.3389/fphar.2024.1379338 (PMC11082339; doi:10.3389/fphar.2024.1379338)
Supplement: Supplementary file 2 [file Table2.docx]

Supplementary Table 2 Univariate Cox regression

|  | Univariate Cox regression |  | *P* for PH assumtpion |
| --- | --- | --- | --- |
|  | HR(95%CI) | *P* |  |
| Group |  |  | 0.073 |
| Non-NDQ | Ref |  |  |
| NDQ | 0.654 (0.489-0.875) | 0.004 |  |
| Age | 0.985 (0.975-0.995) | 0.004 | 0.538 |
| Sex |  |  | 0.702 |
| Male | Ref |  |  |
| Female | 1.189 (0.892-1.584) | 0.237 |  |
| CKD stage |  |  | 0.420 |
| Stage 3 |  |  |  |
| Stage 4 | 3.065 (2.138, 4.396) | 0.000 |  |
| Stage 5 | 6.329 (4.322, 9.366) | 0.000 |  |
| Hb | 0.965 (0.959-0.972) | 0.000 | 0.469 |
| ALB | 0.955 (0.937-0.974) | 0.000 | 0.115 |
| UA | 1.001 (0.999-1.002) | 0.331 | 0.168 |
| Urea | 1.109 (1.088-1.131) | 0.000 | 0.230 |
| TCO2 | 0.866 (0.829-0.906) | 0.000 | 0.286 |
| P | 9.736 (6.275-15.106) | 0.000 | 0.689 |
| Ca^2+^ | 0.064 (0.026-0.160) | 0.000 | 0.158 |
| K^+^ | 2.62 (1.944-3.531) | 0.000 | 0.478 |
| Na^+^ | 0.943 (0.888-1.002) | 0.056 | 0.100 |
| LDL-C | 1.03 (0.922-1.150) | 0.602 | 0.314 |
| TC | 1.029 (0.940-1.126) | 0.539 | 0.252 |
| HDL-C | 0.958 (0.616-1.489) | 0.848 | 0.554 |
| Glu | 1.043 (0.939-1.158) | 0.435 | 0.437 |
| PCR | 1.178 (1.129-1.229) | 0.000 | 0.901 |
| Primary disease diagnoses |  |  | 0.781 |
| Primary glomerulopathy | Ref | 0.221 |  |
| Hypertensive nephropathy | 1.147 (0.608-2.166) | 0.671 |  |
| Diabetic nephropathy | 1.477 (0.765-2.851) | 0.245 |  |
| Other secondary kidney diseases | 0.625 (0.358-1.089) | 0.097 |  |
| Unknown primary diseases | 0.925 (0.654-1.308) | 0.658 |  |
| Hypertension |  |  | 0.134 |
| No | Ref |  |  |
| Yes | 1.467 (1.073-2.005) | 0.016 |  |
| Diabetes |  |  | 0.706 |
| No | Ref |  |  |
| Yes | 1.99 (1.462-2.709) | 0.000 |  |
| Hyperlipidemia |  |  | 0.160 |
| No | Ref |  |  |
| Yes | 1.029 (0.730-1.451) | 0.869 |  |
| Hyperuricemia |  |  | 0.217 |
| No | Ref |  |  |
| Yes | 0.700 (0.510-0.963) | 0.028 |  |
| Anemia |  |  | 0.238 |
| No | Ref |  |  |
| Yes | 3.122 (2.150-4.534) | 0.000 |  |
| CVDs |  |  | 0.249 |
| No | Ref |  |  |
| Yes | 1.223 (0.817-1.830) | 0.327 |  |
| Cerebrovascular diseases |  |  | 0.342 |
| No | Ref |  |  |
| Yes | 1.507 (0.882, 2.574) | 0.133 |  |
| ACEI/ARB |  |  | 0.172 |
| No | Ref |  |  |
| Yes | 0.943 (0.693-1.284) | 0.709 |  |
| Other antihypertensive drugs |  |  | 0.890 |
| No | Ref |  |  |
| Yes | 1.580 (1.187-2.104) | 0.002 |  |
| Hypoglycemic drugs |  |  | 0.983 |
| No | Ref |  |  |
| Yes | 1.742 (1.256-2.416) | 0.001 |  |
| Urate-lowering drugs |  |  | 0.570 |
| No | Ref |  |  |
| Yes | 0.827 (0.593-1.154) | 0.265 |  |
| Lipid-lowering drugs |  |  | 0.517 |
| No | Ref |  |  |
| Yes | 1.433 (1.064-1.930) | 0.018 |  |
| Calcium supplements |  |  | 0.545 |
| No | Ref |  |  |
| Yes | 1.55 0(1.123-2.139) | 0.008 |  |
| Iron supplements |  |  | 0.364 |
| No | Ref |  |  |
| Yes | 2.258 (1.678-3.040) | 0.000 |  |
| Sodium bicarbonate |  |  | 0.261 |
| No | Ref |  |  |
| Yes | 1.415 (1.058-1.891) | 0.019 |  |
| Ketoacid |  |  | 0.441 |
| No | Ref |  |  |
| Yes | 1.964 (1.474-2.617) | 0.000 |  |
| Diuretics |  |  | 0.007 |
| No | Ref |  |  |
| Yes | 2.039 (1.470-2.826) | 0.000 |  |
| Turbidity-removing Chinese patent medicines |  |  | 0.074 |
| No | Ref |  |  |
| Yes | 1.366 (1.023-1.823) | 0.034 |  |
| Tonifying Chinese patent medicines |  |  | 0.570 |
| No | Ref |  |  |
| Yes | 0.886 (0.618-1.271) | 0.511 |  |
| Other Chinese patent medicines |  |  | 0.926 |
| No | Ref |  |  |
| Yes | 0.874 (0.543-1.405) | 0.577 |  |

Note:Hemoglobin, Hb; albumin, ALB; Uric acid, UA; Blood urea nitrogen, Urea; Total carbon dioxide, TCO2; Phosphorous, P; Calcium, Ca^2+^; Potassium, K^+^; Sodium, Na^+^; low-density lipoprotein cholesterol, LDL-C; total cholesterol, TC; high-density lipoprotein cholesterol,HDL-C; Fasting blood glucose, Glu; Protein/creatinine ratio, PCR; Primary Glomerulonephritides included chronic nephritis, nephropathy syndrome and IgA nephropathy.Other secondary nephrosis included systemic lupus erythematosus nephritis, Henoch-Schonlein purpura,Hepatitis B virus-associated nephritis and obstructive nephropathy, etc.;Cardiovascular diseases, CVDs; Angiotensin-converting enzyme inhibitors, ACEIs; Angiotensin receptor blockers, ARBs.
